# Association analysis of polymorphisms rs12997 in *ACVR1* and rs1043784 in *BMP6* genes involved in bone morphogenic protein signaling pathway in primary angle-closure and pseudoexfoliation glaucoma patients of Saudi origin

**DOI:** 10.1186/s12881-020-01076-0

**Published:** 2020-07-08

**Authors:** Altaf A. Kondkar, Tahira Sultan, Taif A. Azad, Essam A. Osman, Faisal A. Almobarak, Saleh A. Al-Obeidan

**Affiliations:** 1 Department of Ophthalmology, College of Medicine, King Saud University, P.O. Box 245, Riyadh, 11411 Saudi Arabia; 2 Glaucoma Research Chair in Ophthalmology, College of Medicine, King Saud University, Riyadh, Saudi Arabia

**Keywords:** Activin, Transforming growth factor-β, Bone morphogenic protein, Glaucoma, Saudi Arabia, Signaling pathway

## Abstract

**Background:**

Glaucoma is a polygenic neurodegenerative disease and the second most common cause of blindness in Saudi Arabia. To test the hypothesis that genetic variants in the genes involved in the bone morphogenic protein (BMP) signaling pathway may be associated with glaucoma, we investigated the association between 3′ untranslated region variants, rs12997 in *ACVR1* and rs1043784 in *BMP6*, and primary angle-closure glaucoma (PACG) and pseudoexfoliation glaucoma (PXG).

**Methods:**

In a case-control study, TaqMan® real-time PCR-based genotyping was done in 444 subjects consisting of 250 controls, 101 PACG and 95 PXG cases, and tested for genetic association with glaucoma-types and other clinical phenotypes.

**Results:**

Rs12997[G] allele in *ACVR1* exhibited significant 2-fold increased risk of PACG (*p* = 0.005) in women but not in men. Similarly, genotype analysis also showed that subjects carrying rs12997[G/G] genotype were at > 2-fold risk of PACG that remained significant after adjustment for age, sex, and Bonferroni correction in the recessive model. Furthermore, this effect was also significant in women only. In PXG, the rs12997[G/G] genotype showed a significant trend towards increased risk of the disease (OR = 2.04, 95% CI = 0.99–4.18, *p* = 0.049) but did not survive the Bonferroni correction. Regression analysis showed that rs12997[G/G] genotype was a significant predictor of PACG independent of age, sex, and rs1043784 genotypes. Likewise, age and rs12997[G/G] genotype showed significant effect on PXG outcome. The rs12997[A/G] genotype showed significant association with cup/disc ratio as compared to wild-type (*p* = 0.005) in PXG. Genotype and allele frequencies of rs1043784 in *BMP6* did not show any significant association either with PACG or PXG.

**Conclusions:**

Our results suggest that the polymorphism rs12997 in the *ACVR1* gene involved in the BMP signaling pathway is significantly associated with PACG and PXG in a Saudi cohort. This is the first study to associate this variant/gene with PACG and PXG. However, further studies would be needed to replicate these findings in a large population-based cohort.

## Background

Obstruction in the outflow pathway of the aqueous humor due to structural changes in the trabecular meshwork (TM) and the resultant elevated intraocular pressure (IOP) are believed to be one of the major contributing factors leading to the damage of the optic nerve head (ONH), retinal ganglion cell (RGC) death and subsequent loss of vision in glaucoma [1]. The precise molecular mechanisms or genetic factors responsible for TM alteration and outflow resistance leading to glaucomatous damage to the eye are still unclear. Recent studies suggest an essential role of growth factors in maintaining ocular homeostasis in tissues related to glaucoma, and alterations in growth factor or their receptors may induce structural or functional changes in the TM or ONH and thereby play an important role in the pathogenesis of glaucoma [2, 3].

Members of the transforming growth factor-β (TGF-β) superfamily of growth factors have been implicated in glaucoma pathogenesis [4–6]. Recent genome-wide association study (GWAS) reported three loci containing genes (*CDKN2B-AS1*, *TGFBR3-CDC7* and *FNDC3B*) linked to POAG which may contribute to the regulation of the TGF-β signaling [7]. Besides TGF-β, the members of the TGF-β family also include bone morphogenetic proteins (BMPs), activins and other signaling molecules [8].

BMPs are known to control a variety of biological functions in the cells [9]. Like the TGF-β cytokines, BMPs induce signaling by binding to the cell surface BMP type I and type II serine/threonine kinase receptors. Following ligand binding, the type II BMP receptor phosphorylates the type I BMP receptor to initiate downstream BMP signaling via Smad or non-Smad pathway to regulate transcription of target genes [9]. Activin A receptor type I (*ACVR1*) is a BMP type I receptor of the TGF-β receptor subfamily. Mutations in the *ACVR1* gene are known to cause fibrodysplasia ossificans progressiva (FOP), a genetic disease characterized by progressive heterotopic ossification [10, 11]. Besides, the inactivation or over-activation of these receptors due to genetic variations or altered (mis)expression are also reported to be involved in cardiovascular, reproductive system and cancer [11]. *Acvr1* has also been reported to function as a tumor suppressor gene in the mouse lens [12]. Bone morphogenetic protein 6 (BMP6) is an ACVR1 ligand related to the TGF-β superfamily. A reduction in the levels of BMP6 has been reported in neovascular and early age-related macular degeneration (AMD) [13, 14] In-vitro studies have shown that BMP6 may protect the retinal pigment epithelial (RPE) cells from oxidative stress and apoptosis [14]. Besides, the expression of BMP6 (and activin A) has also been associated with conjunctival scarring [15].

It can thus be speculated that members of the complex TGF-β/BMP signaling pathway may have a role in maintaining TM homeostasis as well as glaucoma pathogenesis [16]. Likewise, any alterations in the expression of genes involved in BMP signaling as a result of genetic variants, may have functional consequences and influence the disease risk [9]. Accordingly, we investigated genetic variations in members of the TGF-β/BMP signaling and their association with primary angle-closure glaucoma (PACG) and pseudoexfoliation glaucoma (PXG). The study focused on two variants in the 3′ untranslated region (UTR), rs12997 in *ACVR1*, and rs1043784 in *BMP6*. The variants rs12997 and rs1043784 located in the 3’UTR region of the *ACVR1* and *BMP6* genes, respectively, and may result in disruption of microRNA (miRNA)/mRNA binding [17], thereby alter their expression and interfere in subsequent downstream processes and influence the disease risk.

## Methods

### Study design and population

A retrospective case-control study was conducted. The study was approved by the Institutional Ethical Committee at College of Medicine (approval number # 08–657) and conformed to the principles of the Declaration of Helsinki. All the participants provided a written informed consent. Patients of Saudi origin with clinical diagnosis of PACG, PXG and normal controls were enrolled at King Abdulaziz University Hospital, Riyadh, Saudi Arabia.

PACG patients (*n* = 101) were diagnosed based on clinical evidence of anatomically closed angle showing the occurrence of appositional or synnechial closure of the anterior chamber angle (at least 270^o^ of the angle is occluded); raised IOP (≥21 mmHg); presence of optic disk damage with cup/disc ratio of at least 0.7 (in at least one eye); and loss of peripheral or advanced visual field [18]. PXG patients (*n* = 95) showed the presence of flaky exfoliation material along the pupil edges or anterior lens capsule, glaucomatous optic neuropathy and associated visual field loss, and high IOP in either or both the eyes as described previously [19]. Patients harboring secondary forms of glaucoma, history of optic neuropathies or visual impairment unrelated to glaucoma, steroid usage, ocular trauma, absence of sufficient fundus visualization for disk assessment, or refusal to enroll were excluded. A group of healthy Saudi Arab subjects (*n* = 250) recruited from our ophthalmology screening clinics served as controls. The participants were: > 40 years of age, with normal IOP, and free from any form of glaucoma on clinical examination. Subjects refusing to participate were excluded.

### Genotyping of rs12997 and rs1043784

TaqMan® assays, C___7545093_10 and C___2064624_20 (Catalog number: 4351379; Applied Biosystems Inc., Foster City, CA, USA) were used to genotype rs12997 and rs1043784, respectively on ABI 7500 Real-Time PCR System (Applied Biosystems) under conditions recommended by the manufacturer [18].

### Statistical analysis

Hardy-Weinberg Equilibrium (HWE), allele, and genotype associations were tested using Chi-square analysis. Besides, Student’s *t*-test (2-groups) and one-way ANOVA (3-groups) was used to compare continuous variables. Regression analysis was performed to test the effects of multiple factors such as age, sex, and genotypes on POAG. Statistical tests were performed using SPSS version 22 (IBM Inc., Chicago, Illinois, USA). Besides, SNPStats online software (https://www.snpstats.net/start.htm) was also utilized for testing genotype associations. A *p* < 0.05 (2-tailed) was considered significant. Bonferroni’s correction was used to adjust for multiple testing and corrected *p*-values *<* 0.01 was considered where applicable.

## Results

### Demographic data distribution

**Fig. 1:**
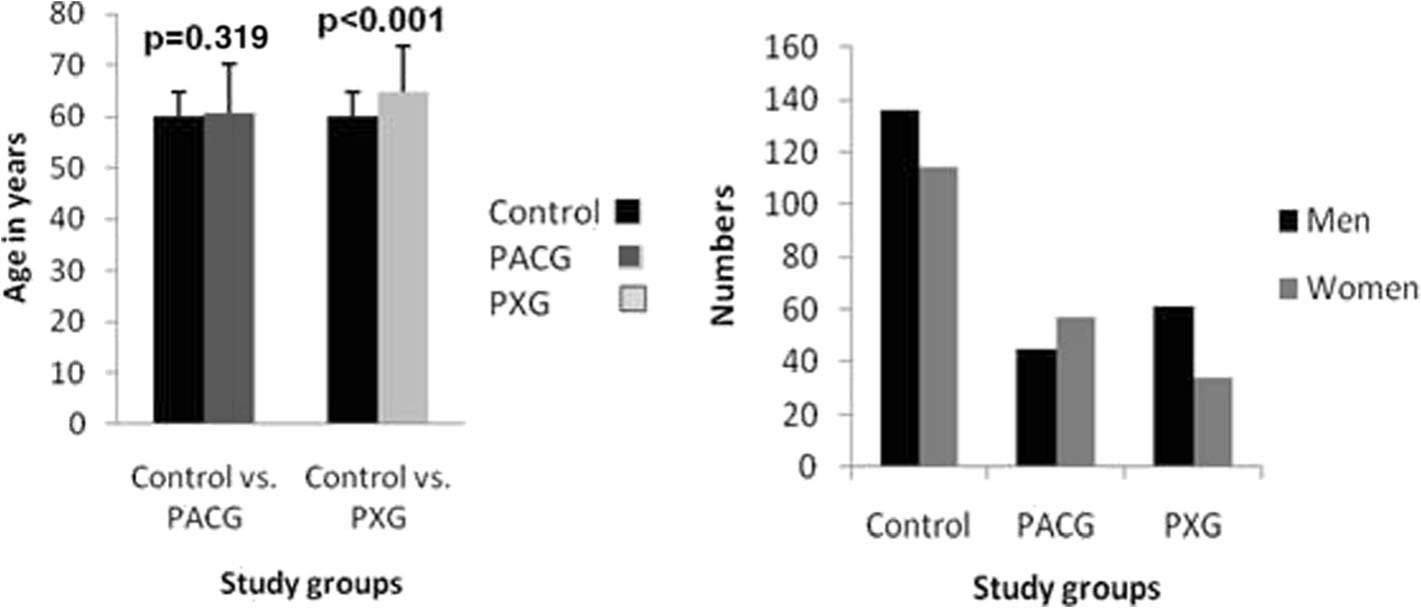
Demographic data distribution of subjects included in this study

The demographic data of subjects included in the study is shown in Fig. 1. The patient groups were found to be older than the control group. The age difference was non-significant in PACG (*p* = 0.319) but significantly older in the PXG (*p* < 0.001) as compared to controls. Similarly, in terms of gender distribution, there was preponderance of men, except in the PACG group. However, the distribution was non-significant in both the PACG (*p* = 0.080) and PXG (*p* = 0.100) groups as compared to controls.

### Allele frequency of rs12997

The distribution of allele frequency of rs12997 according to glaucoma type and gender in cases and controls is shown in Table 1. The control group showed no significant deviation from HWE. As shown in Table 1, the minor allele frequency of rs12997 [G] allele showed a significant trend in PACG (*p* = 0.050), wherein gender-stratification showed that women with G allele exhibited a significant ~ 2-fold increased risk of PACG (*p* = 0.005). Likewise, PXG group also exhibited a significant trend (*p* = 0.056) but did not show any significant gender distribution.

**Table 1:** Minor allele frequency of rs12997[G] polymorphism in cases and controls

Type Group	Cases MAF	Controls MAF	Odds ratio (95% confidence interval)	***p***
PACG				
Total	0.41	0.33	1.40 (0.99–1.96)	**0.050**
Men	0.35	0.36	0.97 (0.58–1.61)	0.920
Women	0.46	0.30	1.93 (1.21–3.07)	**0.005**
PXG				
Total	0.41	0.33	1.39 (0.99–1.97)	**0.056**
Men	0.41	0.36	1.24 (0.89–1.93)	0.329
Women	0.41	0.30	1.61 (0.92–2.82)	0.092

*Abbreviation*: *PACG* primary angle-closure glaucoma, *PXG* pseudoexfoliation glaucoma, *MAF* minor allele frequency

Significant and trend *p*-value in bold

### Allele frequency of rs1043784

The allele frequency distribution of rs1043784 according to glaucoma type and gender in cases and controls is shown in Table 2. In contrast to rs12997, the minor allele [C] of rs1043784 showed no significant distribution either in the PACG (*p* = 0.475) or PXG cases (*p* = 0.446) as compared to controls. Furthermore, gender-stratification also yielded no significant association. Also, there was no significant deviation from HWE.

**Table 2:** Minor allele frequency of rs1043784[C] polymorphisms in cases and controls

Type Group	Cases MAF	Controls MAF	Odds ratio (95% confidence interval)	***p***
PACG				
Total	0.17	0.15	1.17 (0.75–1.82)	0.475
Men	0.19	0.15	1.27 (0.68–2.37)	0.442
Women	0.16	0.14	1.10 (0.59–2.06)	0.751
PXG				
Total	0.17	0.15	1.19 (0.76–1.86)	0.446
Men	0.18	0.15	1.20 (0.68–2.12)	0.360
Women	0.16	0.14	1.14 (0.54–2.39)	0.729

*Abbreviation*: *PACG* primary angle-closure glaucoma, *PXG* pseudoexfoliation glaucoma, *MAF* minor allele frequency

### Genotype analyses of rs12997

The genotype analyses of rs12997 according to glaucoma type and gender-stratification are shown in Tables 3 and 4. Association analysis was performed in co-dominant, dominant, recessive, over-dominant, and log-additive genetic models using SNPStats software.

**Table 3:** Association analysis of rs12997 variant in *ACVR1* with primary angle-closure glaucoma

Group	Genetic Model	Genotype	Control n (%)	PACG n (%)	OR (95% CI)	***p***	AIC	BIC	***p***^**§**^
Overall	Co-dominant	A/A	110 (44.4)	42 (41.6)	1.00	**0.009**	416.5	428.1	**0.02**
		A/G	111 (44.8)	35 (34.6)	0.83 (0.49–1.39)				
		G/G	27 (10.9)	24 (23.8)	**2.33 (1.21–4.48)**				
	Dominant	A/A	110 (44.4)	42 (41.6)	1.00	0.64	423.7	431.4	0.900
		A/G-G/G	138 (55.6)	59 (58.4)	1.12 (0.70–1.79)				
	Recessive	A/A-A/G	221 (89.1)	77 (76.2)	1.00	0.003*	415.1	422.8	**0.009**
		G/G	27 (10.9)	24 (23.8)	**2.55 (1.39–4.69)**				
	Over-dominant	A/A-G/G	137 (55.2)	66 (65.3)	1.00	0.081	420.9	428.6	0.062
		A/G	111 (44.8)	35 (34.6)	0.65 (0.40–1.06)				
	Log-additive	–	–	–	1.36 (0.99–1.89)	0.062	420.4	428.1	0.150
Men	Co-dominant	A/A	55 (41.0)	22 (50.0)	1.00	0.120	200.8	210.4	0.170
		A/G	62 (46.3)	13 (29.6)	0.52 (0.24–1.14)				
		G/G	17 (12.7)	9 (20.4)	1.32 (0.51–3.41)				
	Dominant	A/A	55 (41.0)	22 (50.0)	1.00	0.300	202.0	208.4	0.170
		A/G-G/G	79 (59.0)	22 (50.0)	0.70 (0.35–1.38)				
	Recessive	A/A-A/G	117 (87.3)	35 (79.5)	1.00	0.220	201.6	207.9	0.540
		G/G	17 (12.7)	9 (20.4)	1.77 (0.73–4.32)				
	Over-dominant	A/A-G/G	72 (53.7)	31 (70.5)	1.00	0.048	199.2	205.5	0.060
		A/G	62 (46.3)	13 (29.6)	0.49 (0.23–1.01)				
	Log-additive	–	–	–	0.98 (0.60–1.58)	0.920	203.1	209.4	0.530
Women	Co-dominant	A/A	55 (48.2)	20 (35.1)	1.00	**0.010**	214.5	224	**0.006**
		A/G	49 (43.0)	22 (38.6)	1.23 (0.60–2.53)				
		G/G	10 (8.8)	15 (26.3)	**4.12 (1.60–10.66)**				
	Dominant	A/A	55 (48.2)	20 (35.1)	1.00	0.100	219	225.3	0.140
		A/G-G/G	59 (51.8)	37 (64.9)	1.72 (0.89–3.32)				
	Recessive	A/A-A/G	104 (91.2)	42 (73.7)	1.00	0.003*	212.9	219.1	**0.002**
		G/G	10 (8.8)	15 (26.3)	**3.71 (1.55–8.93)**				
	Over-dominant	A/A-G/G	65 (57.0)	35 (61.4)	1.00	0.58	221.4	227.7	0.420
		A/G	49 (43.0)	22 (38.6)	0.83 (0.44–1.60)				
	Log-additive	–	–	–	**1.85 (1.17–2.92)**	**0.0076**	214.6	220.8	**0.008**

^§^*p*-value adjusted for age and sex in overall group and by age in men and women groups; *Best-fit model *p*-value. *Abbreviations*: *AIC* Akaike’s information criterion, *BIC* Bayesian information criterion, *OR (95% CI)* Odds ratio (95% confidence interval), *PACG* primary angle-closure glaucoma. Note: Significant odds ratio and *p*-value in bold. Bonferroni corrected *p*-value is 0.01

**Table 4:** Association analysis of rs12997 variant in *ACVR1* with primary pseudoexfoliation glaucoma

Group	Genetic Model	Genotype	Control n (%)	PXG n (%)	OR (95% CI)	***p***	AIC	BIC	***p***^**§**^
Overall	Co-dominant	A/A	110 (44.4)	34 (35.8)	1.00	0.150	407.0	418.6	0.52
		A/G	111 (44.8)	44 (46.3)	1.28 (0.76–2.16)				
		G/G	27 (10.9)	17 (17.9)	**2.04 (0.99–4.18)**^†^				
	Dominant	A/A	110 (44.4)	34 (35.8)	1.00	0.150	406.7	414.4	0.34
		A/G-G/G	138 (55.6)	61 (64.2)	1.43 (0.88–2.33)				
	Recessive	A/A-A/G	221 (89.1)	78 (82.1)	1.00	0.091	405.9	413.6	0.37
		G/G	27 (10.9)	17 (17.9)	1.78 (0.92–3.45)				
	Over-dominant	A/A-G/G	137 (55.2)	51 (53.7)	1.00	0.800	408.7	416.4	0.7
		A/G	111 (44.8)	44 (46.3)	1.06 (0.66–1.71)				
	Log-additive	–	–	–	1.39 (0.99–1.97)	0.059*	405.2	412.9	0.25
Men	Co-dominant	A/A	55 (41.0)	23 (37.7)	1.00	0.460	246.8	256.6	0.82
		A/G	62 (46.3)	26 (42.6)	1.00 (0.51–1.96)				
		G/G	17 (12.7)	12 (19.7)	1.69 (0.70–4.09)				
	Dominant	A/A	55 (41.0)	23 (37.7)	1.00	0.660	246.1	252.7	0.83
		A/G-G/G	79 (59.0)	38 (62.3)	1.15 (0.62–2.14)				
	Recessive	A/A-A/G	117 (87.3)	49 (80.3)	1.00	0.210	244.8	251.3	0.53
		G/G	17 (12.7)	12 (19.7)	1.69 (0.75–3.79)				
	Over-dominant	A/A-G/G	72 (53.7)	35 (57.4)	1.00	0.630	246.1	252.6	0.85
		A/G	62 (46.3)	26 (42.6)	0.86 (0.47–1.59)				
	Log-additive	–	–	–	1.24 (0.80–1.90)	0.340	245.4	252	0.64
Women	Co-dominant	A/A	55 (48.2)	11 (32.4)	1.00	0.230	162.5	171.5	0.45
		A/G	49 (43.0)	18 (52.9)	1.84 (0.79–4.27)				
		G/G	10 (8.8)	5 (14.7)	2.50 (0.71–8.76)				
	Dominant	A/A	55 (48.2)	11 (32.4)	1.00	0.098	160.8	166.8	0.22
		A/G-G/G	59 (51.8)	23 (67.7)	1.95 (0.87–4.37)				
	Recessive	A/A-A/G	104 (91.2)	29 (85.3)	1.00	0.330	162.6	168.6	0.54
		G/G	10 (8.8)	5 (14.7)	1.79 (0.57–5.66)				
	Over-dominant	A/A-G/G	65 (57.0)	16 (47.1)	1.00	0.310	162.5	168.5	0.38
		A/G	49 (43.0)	18 (52.9)	1.49 (0.69–3.22)				
	Log-additive	–	–	–	1.64 (0.92–2.93)	0.091	160.7	166.7	0.22

*Abbreviations*: *AIC* Akaike’s information criterion, *BIC* Bayesian information criterion, *OR (95% CI)* Odds ratio (95% confidence interval), *PXG* pseudoexfoliation glaucoma. Note: Significant odds ratio and *p*-value (trend) in bold. Bonferroni corrected *p*-value is 0.01

^§^*p*-value adjusted for age and sex in overall group and by age in men and women groups; ^†^A/A vs G/G *p*-value = **0.049**; *Best-fit model *p*-value

As shown in Table 3, rs12997 polymorphism showed a significant association with PACG in co-dominant and recessive models. The recessive model was observed to be the best-fit as indicated by the Akaike’s information criterion and Bayesian information criterion values. Subjects carrying G/G genotype were at significantly increased (> 2-fold) risk of glaucoma. The effect remained significant after adjustment for age, sex, and Bonferroni correction (p_correction_ = 0.05/5 = 0.01) in the recessive model. However, the significance was lost after Bonferroni correction in the co-dominant model. Furthermore, the association was significant in women but not in men, and the p-value showed significant association even after adjustment for age and multiple testing (Table 3).

Analysis of rs12997 in PXG patients showed a trend towards association as compared to controls (Table 4) with G/G genotype resulting in a 2-fold increased of disease (*p* = 0.049) in co-dominant model. No gender-specific association was observed with PXG (Table 4). However, the trend did not survive the Bonferroni correction.

### Genotype analyses of rs1043784

Rs1043784 variant did not show any significant association with PACG and PXG (Table 5). Besides, a genotype analysis of rs1043784 in PACG and PXG cases in comparison to controls according to gender also did not reveal any gender-specific association in any of the tested genetic models (*data not shown*).

**Table 5:** Association analysis of rs1043784 variant in *BMP6* in cases and controls according to glaucoma types

Group	Genetic Model	Genotype	Control n (%)	Cases n (%)	OR (95% CI)	***p***	AIC	BIC	***p***^**§**^
PACG	Co-dominant	T/T	184 (73.6)	71 (69.6)	1.00	0.75	429.2	440.8	0.73
		C/T	57 (22.8)	27 (26.5)	1.23 (0.72–2.09)				
		C/C	9 (3.6)	4 (3.9)	1.15 (0.34–3.86)				
	Dominant	T/T	184 (73.6)	71 (69.6)	1.00	0.45	427.2	434.9	0.66
		C/T-C/C	66 (26.4)	31 (30.4)	1.22 (0.73–2.02)				
	Recessive	T/T-C/T	241 (96.4)	98 (96.1)	1.00	0.89	427.8	435.5	0.63
		C/C	9 (3.6)	4 (3.9)	1.09 (0.33–3.63)				
	Over-dominant	T/T-C/C	193 (77.2)	75 (73.5)	1.00	0.47	427.2	435	0.5
		C/T	57 (22.8)	27 (26.5)	1.22 (0.72–2.07)				
	Log-additive	–	–	–	1.16 (0.76–1.76)	0.5	427.3	435	0.85
PXG	Co-dominant	T/T	184 (73.6)	64 (67.4)	1.00	0.29	409.6	421.1	0.24
		C/T	57 (22.8)	29 (30.5)	1.46 (0.86–2.48)				
		C/C	9 (3.6)	2 (2.1)	0.64 (0.13–3.04)				
	Dominant	T/T	184 (73.6)	64 (67.4)	1.00	0.25	408.8	416.5	0.24
		C/T-C/C	66 (26.4)	31 (32.6)	1.35 (0.81–2.26)				
	Recessive	T/T-C/T	241 (96.4)	93 (97.9)	1.00	0.46	409.5	417.2	0.38
		C/C	9 (3.6)	2 (2.1)	0.58 (0.12–2.72)				
	Over-dominant	T/T-C/C	193 (77.2)	66 (69.5)	1.00	0.14	407.9	415.6	0.13
		C/T	57 (22.8)	29 (30.5)	1.49 (0.88–2.52)				
	Log-additive	–	–	–	1.18 (0.76–1.83)	0.46	409.5	417.2	0.45

*Abbreviations*: *OR (95% CI)* Odds ratio (95% confidence interval), *AIC* Akaike’s information criterion, *BIC* Bayesian information criterion, *PACG* primary angle-closure glaucoma, *PXG* pseudoexfoliation glaucoma

^§^*p*-value adjusted for age and sex in overall group and by age in men and women groups

### Effect of age, sex, and genotypes on disease outcome

Table 6 shows the results of regression analysis of age, sex, rs12997, and rs1043784 variants on glaucoma outcomes. The regression analysis revealed that rs12997 and G/G genotype was a significant predictor of PACG independent of age, sex, and rs1043784 genotypes. Likewise, age, rs12997 and G/G genotype showed significant effect on PXG outcome.

**Table 6:** Regression analysis to determine the effect age, sex and polymorphisms on glaucoma risk

Group Variables	B	SE	Wald	Odds ratio (95% confidence interval)	***p***
**PACG**					
Age	0.016	0.016	0.995	1.02 (0.98–1.04)	0.319
Sex	−0. 467	0.255	3.371	0. 62 (0.38–1.03)	0. 066
Rs12997			7.904		**0.019**
A/G	−0. 262	0. 281	0. 870	0.77 (0.44–1.33)	0.351
G/G	0.748	0.354	4.457	2.11 (1.05–4.23)	**0.035**
Rs1043784			0.675		0.714
T/C	0.185	0.297	0.389	1.20 (0.67–2.15)	0.533
C/C	−0.329	0.709	0.216	0.72 (0.18–2.88)	0.642
Constant	−1.498	0.961	2.43	0.223	0.119
**PXG**					
Age	0.075	0.017	20.488	1.08 (1.04–1.11)	**0.000**
Sex	−0.299	0.278	1.159	1.34 (0.78–2.32)	0.282
Rs12997			6.227		**0.044**
A/G	0.188	0.295	0.404	1.20 (0.67–2.15)	0.525
G/G	1.033	0.418	6.107	2.81 (1.23–6.37)	**0.013**
Rs1043784			2.932		0.231
T/C	0.455	0.303	2.26	1.57 (0.87–2.85)	0.133
C/C	−0.559	0.854	0.427	0.57 (0.10–3.05)	0.513
Constant	−6.028	1.095	30.318	0.002	0.000

*Abbreviations*: *PACG* primary angle-closure glaucoma, *PXG* pseudoexfoliation glaucoma

Significant *p*-value in bold

### Effect on genotypes on clinical indices of glaucoma

**Fig. 2:**
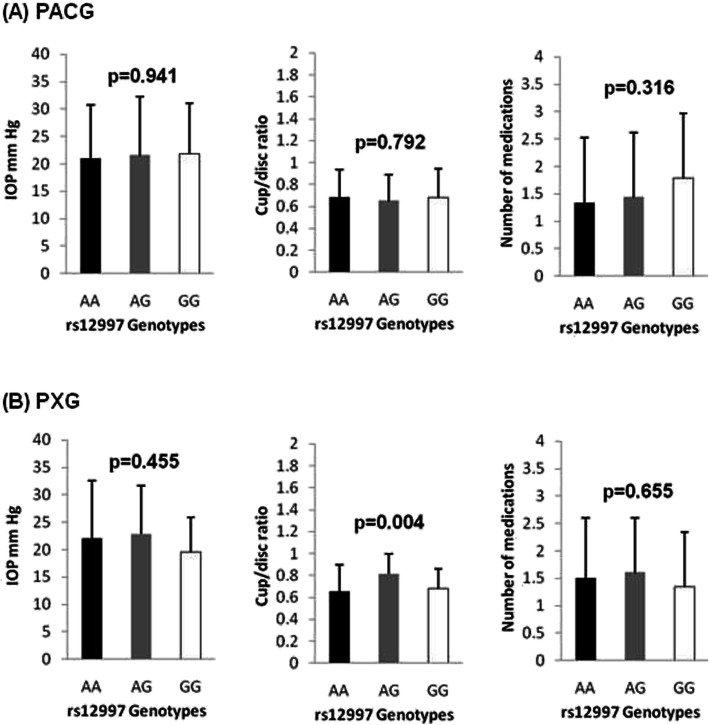
Genotype effects of rs12997 variant on intraocular pressure (IOP), cup/disc ratio and number of antiglaucoma medication in (**a**) PACG and (**b**) PXG patient groups

**Fig. 3:**
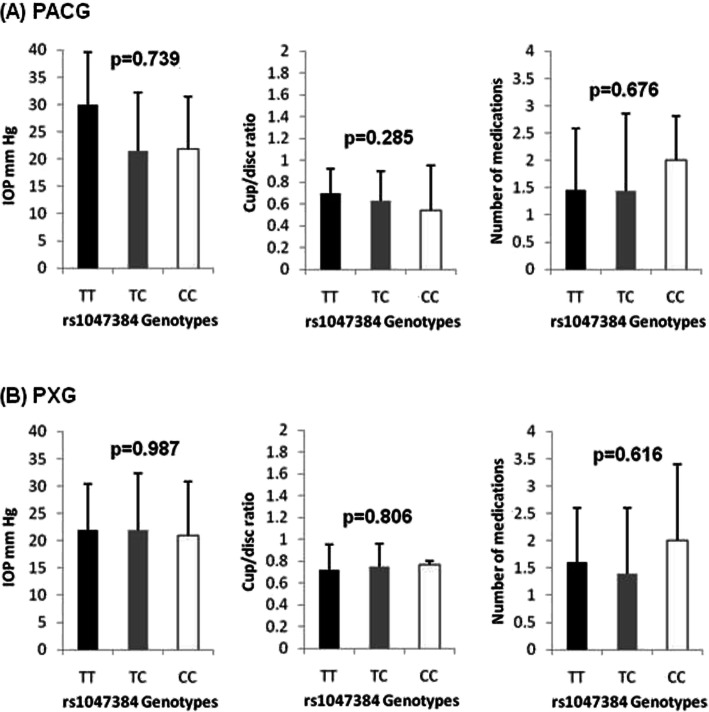
Genotype effects of rs1043784 variant on intraocular pressure (IOP), cup/disc ratio and number of antiglaucoma medication in (**a**) PACG and (**b**) PXG patient groups

Figure 2 shows the genotype effect of rs12997 on IOP, cup/disc ratio and number of antiglaucoma medications in the PACG and PXG groups. These are clinical indicators related to the severity of the disease. The distribution showed no significant effect on any of these phenotypes, except for cup/disc ratio in PXG cases (*p* = 0.004). Post-hoc analysis showed that A/G genotype exhibited increased cup/disc ratio as compared to A/A (wild-type) genotype (*p* = 0.005). Figure 3 shows the genotype effect of rs1043784 on IOP, cup/disc ratio and number of antiglaucoma medications in the PACG and PXG groups. The genotype distribution showed no significant effect on any of these phenotypes in both the PACG and PXG patient groups.

## Discussion

In the present study, we report a previously unreported association between variant rs12997 in the *ACVR1* gene involved in BMP signaling and patients with PACG and PXG in a Saudi cohort.

The regulation of *ACVR1* (also known as *ALK2*) gene expression is still not completely understood. ACVR1 can function via the TGF-β/BMP signaling through a variety of different mechanisms. Mutations in *ACVR1* have been reported to show increased responsiveness to certain BMP ligand activation in a variety of cell types, leading to over-activation of *ACVR1* and dysregulated BMP signaling [20–22]. Activins can also interact with ACVR1, and compete with BMP ligands [23]. The competition for ligand may cause dysregulated signaling outcomes due to any imbalance in the levels of epxression between type I and type II BMP receptors [24]. Besides, abnormal ACVR1 activity is also reported to be proinflammatory, causing altered immune function via NF-κB and p38MAPK activity signaling leading to pathological outcomes [25]. Besides, association of genetic variants in *ACVR1* has been reported in breast cancer [26], and with anti-Mullerian hormone level in women having polycystic ovary syndrome [27].

Interestingly, ACVR1 has been reported to function as a critical regulator of the BMP/Wnt signaling pathway to promote proliferation and metastasis [26, 28]. Genes involved in canonical and non-canonical Wnt signaling pathways have been reported to be expressed in the human TM and the role of the Wnt signaling pathway in the regulation of TM homeostasis and IOP is well documented [29, 30]. Also, over-expression of secreted frizzled-related protein-1 (sFRP-1), a Wnt signaling antagonist, in glaucomatous TM cells has been demonstrated to be responsible for elevated IOP in glaucoma [31]. Similarly, ACVR1 has been shown to inhibit Wnt signaling in osteoblasts by suppressing Wnt inhibitors SOST and DKK1 [32]. Likewise, *Acvr1*-deficiency (loss-of-function) was found to increase osteogenesis by activating Wnt signaling and decreasing the expression levels of these Wnt inhibitors [32]. Based on these studies, it can be speculated there is a plausible role for ACVR1 in TM modulation and IOP regulation via Wnt signaling regulation.

PACG is a more common form of glaucoma in Asia [33, 34], involving anatomical obstruction of the outflow pathway [1]. Likewise, PXG is a severe form of open-angle glaucoma associated with worse prognosis. PXG is characterized by the abnormal deposition of pseudoexfoliative material (fibrillar extracellular matrix (ECM)) in the anterior segment of the eye causing the obstruction [35]. It is difficult to elucidate the precise mechanism(s) by which the variant rs12997 in *ACVR1* may influence the risk of these glaucoma types. Nevertheless, rs12997 is located in the 3’UTR region of the gene, which are known to regulate mRNA stability. The functional analysis of the 3’UTR region of *ACVR1* has reported the involvement of specific miRNAs in regulating its gene expression [36]. It has been observed that rs12997 variation in *ACVR1* may affect the binding of miR-330-3p and result in loss of *ACVR1* regulation [17]. Hence, *ACVR1* with G allele may result in its over-expression as compared to the wild-type allele, thereby showing an association with PACG and PXG by mechanism(s) regulating the ACVR1/BMP/Wnt signaling pathway as described above. Besides, Wnt signaling pathway play a key role in ECM cell behaviour and elasticity [37], and studies have demonstrated a link between Wnt antagonism and increased TM stiffness that may contribute to glaucoma progression [38]. It is thus possible that over-expression of *ACVR1* may result in dysregulated Wnt signaling causing ECM abnormalities in the TM and elevated IOP. Further in-vitro and molecular studies are needed to support this hypothesis.

Our study showed no association between variant rs1047384 in *BMP6* and PACG and PXG, indicating BMP6 may not have a major role in glaucoma pathogenesis. However, *BMP6* locus has been associated with lung function in a GWAS [39]. Likewise, animal studies have provided evidence for a developmental role of BMP4 and/or TGF-β2 in mesenchyme morphogenesis in the anterior eye [40]. Thus the role of other variants in *BMP6* or other BMPs in glaucoma cannot be ruled out and needs further investigations [41].

The results of this study need cautious interpretation due to its certain limitations. The study examined a relatively small sample size. On the basis of MAF observed in our study population and assuming an OR of 2.0 (α = 0.05), the study exhibited a power of 0.82 and 0.80 for rs12997 association in PACG and PXG, respectively. Likewise, variant rs1043784 demonstrated powers of 0.70 and 0.68 to detect association with PACG and PXG, respectively. However, to detect an effect of 1.5 or less, as is most commonly seen in genetic association investigations, a multicenter study with larger sample-size needs to be performed to confirm these results. Besides, our results do not provide any functional/mechanistic evidence for the role of rs12997 in *ACVR1* in PACG and PXG. Furthermore, linkage with other causal variant(s), gene-gene or gene-environment cannot be ruled out.

## Conclusions

In conclusion, our study reports for the first time that genetic variant rs12997 in the *ACVR1* gene is associated with PACG and PXG, and suggests that *ACVR1*, a member of the BMP signaling pathway may play an important role in the complex pathogenesis of these diseases. However, as pointed out earlier, our results need to be replicated in other cohorts of different ethnicity and in a large population-based sample size to draw definite conclusions and evaluate the utility of this variant/gene as a potential genetic biomarker in glaucoma. Besides, based on the reports of miRNA-based regulation of *ACVR1* [26, 36], understanding the underlying mechanisms of the *ACVR1* gene biology may offer potential therapeutic drug target in glaucoma.

## Data Availability

The datasets used and/or analysed during the current study are available from the corresponding author on reasonable request.

## Abbreviations

ACVR1: Activin A receptor type I
AMD: Age-related macular degeneration
BMP: Bone morphogenic proteins
BMP6: Bone morphogenetic protein 6
CI: Confidence interval
ECM: Extracellular matrix
FOP: Fibrodysplasia ossificans progressive
HWE: Hardy-Weinberg equilibrium
IOP: Intraocular pressure
miRNA: MicroRNA
OR: Odds ratio
ONH: Optic nerve head
PACG: Primary angle-closure glaucoma
PXG: Pseudoexfoliation glaucoma
RGC: Retinal ganglion cell
RPE: Retinal pigment epithelia
TM: Trabecular meshwork
TGF-β: Transforming growth factor-β
UTR: Untranslated region

## References

[1] Weinreb RN, Aung T, Medeiros FA (2014). The pathophysiology and treatment of glaucoma: a review. JAMA.

[2] Wordinger RJ, Fleenor DL, Hellberg PE, Pang IH, Tovar TO, Zode GS (2007). Effects of TGF-beta2, BMP-4, and gremlin in the trabecular meshwork: implications for glaucoma. Invest Ophthalmol Vis Sci.

[3] Browne JG, Ho SL, Kane R, Oliver N, Clark AF, O’Brien CJ (2011). Connective tissue growth factor is increased in pseudoexfoliation glaucoma. Invest Ophthalmol Vis Sci.

[4] Fuchshofer R, Birke M, Welge-Lussen U, Kook D, Lutjen-Drecoll E (2005). Transforming growth factor-beta 2 modulated extracellular matrix component expression in cultured human optic nerve head astrocytes. Invest Ophthalmol Vis Sci.

[5] Robertson JV, Golesic E, Gauldie J, West-Mays JA (2010). Ocular gene transfer of active TGF-beta induces changes in anterior segment morphology and elevated IOP in rats. Invest Ophthalmol Vis Sci.

[6] Prendes MA, Harris A, Wirostko BM, Gerber AL, Siesky B (2013). The role of transforming growth factor beta in glaucoma and the therapeutic implications. Br J Ophthalmol.

[7] Li Z, Allingham RR, Nakano M, Jia L, Chen Y, Ikeda Y (2015). A common variant near TGFBR3 is associated with primary open angle glaucoma. Hum Mol Genet.

[8] Katagiri T, Watabe T. Bone Morphogenetic Proteins. Cold Spring Harb Perspect Biol. 2016;8(6):a021899. 10.1101/cshperspect.a021899 PMC4888821 27252362

[9] Gomez-Puerto MC, Iyengar PV, Garcia de Vinuesa A, Ten Dijke P, Sanchez-Duffhues G (2019). Bone morphogenetic protein receptor signal transduction in human disease. J Pathol.

[10] Bocciardi R, Bordo D, Di Duca M, Di Rocco M, Ravazzolo R (2009). Mutational analysis of the ACVR1 gene in Italian patients affected with fibrodysplasia ossificans progressiva: confirmations and advancements. Eur J Hum Genet.

[11] Valer JA, Sanchez-de-Diego C, Pimenta-Lopes C, Rosa JL, Ventura F (2019). ACVR1 function in health and disease. Cells.

[12] Wiley LA, Rajagopal R, Dattilo LK, Beebe DC (2011). The tumor suppressor gene Trp53 protects the mouse lens against posterior subcapsular cataracts and the BMP receptor Acvr1 acts as a tumor suppressor in the lens. Dis Model Mech.

[13] Hadziahmetovic M, Song Y, Wolkow N, Iacovelli J, Kautz L, Roth MP (2011). Bmp6 regulates retinal iron homeostasis and has altered expression in age-related macular degeneration. Am J Pathol.

[14] Chen L, Liu M, Luan Y, Liu Y, Zhang Z, Ma B (2018). BMP6 protects retinal pigment epithelial cells from oxidative stressinduced injury by inhibiting the MAPK signaling pathways. Int J Mol Med.

[15] Andreev K, Zenkel M, Kruse F, Junemann A, Schlotzer-Schrehardt U (2006). Expression of bone morphogenetic proteins (BMPs), their receptors, and activins in normal and scarred conjunctiva: role of BMP-6 and activin-a in conjunctival scarring?. Exp Eye Res.

[16] Tovar-Vidales T, Fitzgerald AM, Clark AF (2016). Human trabecular meshwork cells express BMP antagonist mRNAs and proteins. Exp Eye Res.

[17] Gong J, Shen N, Zhang HM, Zhong R, Chen W, Miao X (2014). A genetic variant in microRNA target site of TGF-beta signaling pathway increases the risk of colorectal cancer in a Chinese population. Tumour Biol.

[18] Abu-Amero KK, Azad TA, Mousa A, Osman EA, Sultan T, Al-Obeidan SA (2013). A catalase promoter variant rs1001179 is associated with visual acuity but not with primary angle closure glaucoma in Saudi patients. BMC Med Genet.

[19] Kondkar AA, Azad TA, Almobarak FA, Kalantan H, Al-Obeidan SA, Abu-Amero KK (2018). Elevated levels of plasma tumor necrosis factor alpha in patients with pseudoexfoliation glaucoma. Clin Ophthalmol.

[20] Shen Q, Little SC, Xu M, Haupt J, Ast C, Katagiri T (2009). The fibrodysplasia ossificans progressiva R206H ACVR1 mutation activates BMP-independent chondrogenesis and zebrafish embryo ventralization. J Clin Invest.

[21] Song GA, Kim HJ, Woo KM, Baek JH, Kim GS, Choi JY (2010). Molecular consequences of the ACVR1(R206H) mutation of fibrodysplasia ossificans progressiva. J Biol Chem.

[22] Billings PC, Fiori JL, Bentwood JL, O'Connell MP, Jiao X, Nussbaum B (2008). Dysregulated BMP signaling and enhanced osteogenic differentiation of connective tissue progenitor cells from patients with fibrodysplasia ossificans progressiva (FOP). J Bone Miner Res.

[23] Olsen OE, Sankar M, Elsaadi S, Hella H, Buene G, Darvekar SR, et al. BMPR2 inhibits activin and BMP signaling via wild-type ALK2. J Cell Sci. 2018;131(11):jcs213512. 10.1242/jcs.213512 29739878

[24] Hatsell SJ, Idone V, Wolken DM, Huang L, Kim HJ, Wang L (2015). ACVR1R206H receptor mutation causes fibrodysplasia ossificans progressiva by imparting responsiveness to activin A. Sci Transl Med.

[25] Barruet E, Morales BM, Cain CJ, Ton AN, Wentworth KL, Chan TV (2018). NF-kappaB/MAPK activation underlies ACVR1-mediated inflammation in human heterotopic ossification. JCI Insight.

[26] Wang Y, Zhang Z, Wang J (2018). MicroRNA-384 inhibits the progression of breast cancer by targeting ACVR1. Oncol Rep.

[27] Kevenaar ME, Themmen AP, van Kerkwijk AJ, Valkenburg O, Uitterlinden AG, de Jong FH (2009). Variants in the ACVR1 gene are associated with AMH levels in women with polycystic ovary syndrome. Hum Reprod.

[28] Li L, Liu Y, Guo Y, Liu B, Zhao Y, Li P (2015). Regulatory MiR-148a-ACVR1/BMP circuit defines a cancer stem cell-like aggressive subtype of hepatocellular carcinoma. Hepatology.

[29] Webber HC, Bermudez JY, Millar JC, Mao W, Clark AF (2018). The role of Wnt/beta-catenin signaling and K-cadherin in the regulation of intraocular pressure. Invest Ophthalmol Vis Sci.

[30] Mao W, Millar JC, Wang WH, Silverman SM, Liu Y, Wordinger RJ (2012). Existence of the canonical Wnt signaling pathway in the human trabecular meshwork. Invest Ophthalmol Vis Sci.

[31] Wang WH, McNatt LG, Pang IH, Millar JC, Hellberg PE, Hellberg MH (2008). Increased expression of the WNT antagonist sFRP-1 in glaucoma elevates intraocular pressure. J Clin Invest.

[32] Kamiya N, Kaartinen VM, Mishina Y (2011). Loss-of-function of ACVR1 in osteoblasts increases bone mass and activates canonical Wnt signaling through suppression of Wnt inhibitors SOST and DKK1. Biochem Biophys Res Commun.

[33] Tham YC, Li X, Wong TY, Quigley HA, Aung T, Cheng CY (2014). Global prevalence of glaucoma and projections of glaucoma burden through 2040: a systematic review and meta-analysis. Ophthalmology.

[34] Al Obeidan SA, Dewedar A, Osman EA, Mousa A (2011). The profile of glaucoma in a tertiary ophthalmic University Center in Riyadh, Saudi Arabia. Saudi J Ophthalmol.

[35] Vazquez LE, Lee RK (2014). Genomic and proteomic pathophysiology of pseudoexfoliation glaucoma. Int Ophthalmol Clin.

[36] Mura M, Cappato S, Giacopelli F, Ravazzolo R, Bocciardi R (2012). The role of the 3’UTR region in the regulation of the ACVR1/Alk-2 gene expression. PLoS One.

[37] Du J, Zu Y, Li J, Du S, Xu Y, Zhang L (2016). Extracellular matrix stiffness dictates Wnt expression through integrin pathway. Sci Rep.

[38] Morgan JT, Raghunathan VK, Chang YR, Murphy CJ, Russell P (2015). Wnt inhibition induces persistent increases in intrinsic stiffness of human trabecular meshwork cells. Exp Eye Res.

[39] Loth DW, Soler Artigas M, Gharib SA, Wain LV, Franceschini N, Koch B (2014). Genome-wide association analysis identifies six new loci associated with forced vital capacity. Nat Genet.

[40] Chang B, Smith RS, Peters M, Savinova OV, Hawes NL, Zabaleta A (2001). Haploinsufficient Bmp4 ocular phenotypes include anterior segment dysgenesis with elevated intraocular pressure. BMC Genet.

[41] Wordinger RJ, Sharma T, Clark AF (2014). The role of TGF-beta2 and bone morphogenetic proteins in the trabecular meshwork and glaucoma. J Ocul Pharmacol Ther.

